# Claw Characteristics of Culled Sows from Three Farrow-to-Finish Greek Farms. Part 1: Claw Length Measurements, Lesion Scores and Their Association

**DOI:** 10.3390/vetsci8070126

**Published:** 2021-07-04

**Authors:** Georgios A. Papadopoulos, Sofia Chalvatzi, Fotios Kroustallas, Vassilis Skampardonis, Mihaela Cernat, Christina Marouda, Vassilios Psychas, Theofilos Poutahidis, Leonidas Leontides, Paschalis Fortomaris

**Affiliations:** 1 Laboratory of Animal Husbandry, Faculty of Veterinary Medicine, School of Health Sciences, Aristotle University of Thessaloniki, 54124 Thessaloniki, Greece; schalvat@vet.auth.gr (S.C.); fotios2204@yahoo.gr (F.K.); fortomap@vet.auth.gr (P.F.); 2 Department of Epidemiology, Biostatistics and Economics of Animal Production, School of Veterinary Medicine, University of Thessaly, 43132 Karditsa, Greece; bskamp@uth.gr (V.S.); tsernat@uth.gr (M.C.); leoleont@uth.gr (L.L.); 3 Laboratory of Pathology, Faculty of Veterinary Medicine, School of Health Sciences, Aristotle University of Thessaloniki, 54124 Thessaloniki, Greece; cmarouda@vet.auth.gr (C.M.); psychas@vet.auth.gr (V.P.); teoput@vet.auth.gr (T.P.)

**Keywords:** claws, lesion scores, lengths, sows, genetic lines

## Abstract

The aim of the study was to investigate variations in lengths and lesions in claws of culled sows and to evaluate their association. All four feet of 185 sows from three Greek farrow-to-finish farms (Farm A: 57 sows; Farm B: 64 sows; Farm C: 64 sows) were examined for lesions and their lengths were measured. All claw lengths were lower in sows of farm C compared to those from sows of B and A. Claw lengths in sows of farm B were lower compared to those from A for all lateral toes of front feet and for all medial and four out of three lateral toes of rear feet. Sum of length measurements of the main toes of the front feet (SLF) associated with lesions on sole, white line and heel of front feet, while sum of length measurements of the main toes of the rear feet (SLR) associated with all lesions of the rear feet. The lengths of the main toes were correlated with the length of dew claws on front and rear feet. Overall, sows’ claw lesion severity and claw lengths may differ between farms and frequency of lesions is higher in longer claws.

## 1. Introduction

The pig is tetradactylous with two main weight-bearing digits and two accessory ones with no load-carrying capacity [1]. Each digit is surrounded by the claw, which is a keratinized tissue [1]. In sows, claw elements may be subjected to lesions, such as erosion and cracks, while overgrown claws may also be frequent [2]. Lesions are more often on lateral hooves compared to medial ones due to greater weight-bearing capacity [3]. Anatomically, the heel bears most of the weight in the lateral digits of pigs, while in the medial ones the primary usually the tip of the toe bears most of the weight [1]. 

The impaired claw quality has been shown to have significant negative impact on longevity, productive performance and welfare of sows [4,5,6,7,8]. The increased awareness of claw quality importance in sow breeding herds has guided researchers to focus on studying the frequency and severity of hoof growth abnormalities and lesions [8,9,10,11,12,13]. Both visual scoring [7,8,9,10,14] and quantitative measurements [15,16,17,18,19,20,21] of claw lengths have been employed to detect overgrowth. The calculation of a wide range of additional conformational traits such as hoof volume and size, sole area, distal toe angle and toe:heel ratio has also been adopted to provide deeper insight into hoof morphology [20,21]. Recently, studies have been conducted to address the missing information on the rates of formation and loss of horn tissue in hooves of gilts [15], sows [21] and weaned pigs [20]. Qualitative assessment [7,14,15,16,17,19,21] and histopathological characterization [12,13,18] of claw lesions have been also subjects of extensive research.

Based on the published research so far, it is evident that claw lesions are common in modern hyperprolific sows, while also claw lengths may differ between individual sows. Apart from two recent studies [17,19], still it has not yet been extensively investigated the presence or not of an association between lesions and dissimilarity in claw length. It could be hypothesized that longer claws may predispose to a greater risk of damage of claws, and hence to a greater lesion score. Breed differences have been reported for sows and boars regarding certain morphological hoof properties such as claw length, growth and size dissimilarity [2,22,23,24,25]. Still little data are available for differences between sows of common genetic lines. Thus, the present study was undertaken in order (i) to detect possible variations in claw lengths (dorsal, diagonal, heel–sole and dew claw lengths) and lesion scores in culled multiparous sows of the three commercial farms with different genetic lines and (ii) to describe a possible association between claw lengths and lesion severity.

## 2. Materials and Methods

### 2.1. Participating Farms

Three Greek farrow to finish farms were included in the study. The experimental period started on January 2019 and ended on April 2020. The herds complied with the EU directive 2001/88/DC on animal welfare. The sow capacity of the participating farms was 250 (Farm A), 350 (Farm B) and 370 sows (Farm C). The farms were situated in the Larissa prefecture-Periphery of Thessaly (Farms A and B) and in Kozani prefecture-Periphery of Western Macedonia (Farm C). The collaborating farms were participants together with the academic units of the “T1EDK -02073- FITSOW’ research project investigating the longevity and welfare of sows in commercial Greek farms (financed by the European Regional Development Fund of the European Union and by Greek national funds). The sows of each farm belonged to different genetic lines (Farm A: PIC; Farm B: Danbred; Farm C: Topigs). All sows’ diets in the three participating farms were supplemented with the same premix containing the same quantity of chelated minerals, in order to minimize any effects on claw characteristics due to differences in mineral supplementation. The participating farms were using the same premixes for the sows’ diets for at least a year prior the collection of samples. Sows were housed on fully slatted concrete floors during the gestation period and on fully slatted plastic floors during the farrowing period. Sow culling was decided by each farm manager and there was no involvement of members of our research team on sow removals. The vast majority of sows were culled due to old age. Other reasons of sows’ removal were leg or reproductive problems. Members of the research team were informed two to three days in advance by email or by phone by the farmers before culled sows were sent to collaborating slaughterhouses in order to fine-tune sample collection and transfer within the same day to the Laboratory of Pathology of the Veterinary Faculty of Aristotle University of Thessaloniki for further evaluation. 

### 2.2. Slaughterhouse Sampling Protocol

The Council Directive 93/119/EC on the protection of animals at the time of slaughter or killing was applied for all animals included in the study. Sow’s parity ranged from 0 to 7 (median 5) in Farm A, from 0 to 9 (median 6) in Farm B and from 0 to 8 (median 6) in Farm C. Sows were slaughtered separately than the rest pigs on each sampling day, giving us sufficient time to acquire all 4 feet of each sow and collect also the respective ear tag. Subsequently, all four feet together with the sow’s identification number (eartag) were placed in a plastic bag. The samples were then stored in polystyrene boxes with ice-packs and transferred to the Laboratory of Pathology of the Veterinary Faculty, Aristotle University. All four feet of 185 culled sows from three Greek farrow to finish herds (Herd A: 57 sows; Herd B: 64 sows; Herd C: 64 sows) were collected.

### 2.3. Macroscopical Examination of Sow Claw Lesions

The medial and lateral claws of front and rear sows’ feet were macroscopically examined for lesions and scored by two of us (VP, CM). The evaluation and the scoring of the intensity of lesions in the anatomical sites of the claw adopted by Lisgara et al. [9] is given in Table 1. Both assessors were trained veterinarians and members of the Laboratory of Pathology of the Veterinary Faculty of Aristotle University. Additionally, they were familiar with the scoring system as they have applied it in previous research studies. To eliminate inter-observer reliability both assessors evaluated the samples simultaneously and recorded a single score based on the scoring scheme used.

Additionally, claw length measurements were collected for all claws and dew claws, using digital calipers (Facom 150 mm Digital Caliper 0.01 mm). The following lengths were measured per toe: (A) the dorsal hoof length (along dorsal wall from just below the coronary band to the end of the wall), (B) the diagonal hoof length (along abaxial wall from the bottom of the wall at the toe to the top of abaxial wall-heel junction), (C) the heel–sole length, which was the length of the abaxial wall (sole) and bulb (heel) that are in contact with the floor surface along ventral surface from the top of the toe to the caudal end of the heel and (D) the dewclaw length (along dorsal wall from just be-low the coronary band to the end of the wall). Based on these measurements, the difference between the dorsal lengths of lateral and medial were calculated.

### 2.4. Statistical Analysis

All statistical analyses were performed using Stata 13.1 (Stata Statistical Software, College Station, TX, USA) and evaluated at the 5% level of significance. Initially, differences for the measured claw lengths were evaluated among herds by one-way ANOVA. Multiple comparisons were done with Tukey’s test. Statistical significance was considered at *p* < 0.05. Results on claw lengths were presented as mean ± standard deviation (SD). Scoring of lesions at the five anatomical sites of the claw and length measurements, resulted in forty (5 sites per claw × 8 toes = 40) and thirty-two (3 measurements × 8 claws + 8 dew claws = 32) variables, respectively, from each sampled sow. Multicollinearity was a major issue in the analysis of these data. A high correlation between these variables was noted as they constituted measurements of the same subject, foot or claw [9]. In such cases, Dohoo et al. [26] proposed either the creation of scores or indices which combine data from several independent variables, or employment of multivariable methods such as principal component analysis or factor analysis. Therefore, regarding claw length measurements four scores were created: (i) a score for the sum of length measurements of the main toes of the front feet which was created by adding the measurements A, B and C of the four front main toes (SLF), (ii) a score for the sum of length measurements of the main toes of the rear feet which was created by adding the measurements A, B and C of the four rear main toes (SLR), (iii) a score for the sum of the length measurements of the four dewclaws of the front feet (SDCF) and a score for the sum of the lengths of the four dewclaws of the rear feet (SDCR). It was documented in previous research that there was a difference between the frequency of claw overgrowth and lesions in the rear compared to those in the front feet [15,19]. For this reason, different sums for front and rear were created. Pearson’s correlation coefficient was used to investigate the association between the length scores of the main (SLF and SLR) and dew claws (SDCF and SDCR) on front and rear feet. To consolidate the available information contained in all lesion scores into a new smaller set of uncorrelated variables and simultaneously allow for the detection of a possible discerned or combined effect of claw lesions according to their anatomical location, we performed factor analysis [9]. In this analysis, the original claw lesion score variables were considered as a linear combination of the produced factor loadings plus an error term [26]. Two separate factor analyses were performed, one for claw lesion scores of the front and another for claw lesion scores of the rear feet. More specifically, factors were extracted by the iterated principal-factor method, accounting for higher total variance [27,28]. Suitability of individual variables for inclusion and use in factor analysis was determined by the Kaiser–Meyer–Olkin measure of adequacy. According to Berghaus et al. [27], we resolved in the number of factors to maintain for interpretation by compromising parsimony, interpretability and the total amount of variation in the original variables that was explained by the factors in the model. The factors to retain for interpretation were determined according to Kaiser’s criterion (initial eigenvalue ≥ 1), a Scree-test plot and the number of factors that are needed to account for a certain proportion of the variance detected in the original variables [29]. Orthogonal and oblique factor rotations were also evaluated, but ultimately an orthogonal rotation was selected for the final analysis because it resulted in a relatively simple and interpretable structure while maintaining factor independence [27]. For the interpretation of rotated factors |Factor loadings| > 0.40 were used [27]. For the factor analysis of claw lesion scores of the front feet, four factors had an eigenvalue ≥ 1, suggesting that they should be retained for interpretation according to Kaiser’s criterion and further validation of the Screeplot method. After consideration of the amount of variance explained, we retained these four factors, cumulatively accounting for almost 75 percent of the variance in the original variables. For the factor analysis of claw lesion scores of the rear feet, four factors had an eigenvalue ≥ 1. After, co-evaluation of Kaiser’s criterion, the Scree plot method and the amount of explained variance, we retained 5 factors, cumulatively accounting for almost 77 percent of the variance in the original variables. Subsequently, in both cases the regression method was used to produce standardized factor scores for the retained factors [27]. 

Subsequently, the produced standardized factor scores for the front and rear feet were evaluated as possible predictors of SLF and SLR in two separate multiple linear regression models. The initial models included the produced standardized factor scores, a dummy variable coding for the farm of sow origin to control for unmeasured management factors operating at the herd level (e.g., different genotypes) and the sows’ parity which accounted for the documented effect of sow’s age in the occurrence and severity of claw lesions and toe overgrowths [9]. The initial models were subsequently reduced in a stepwise fashion until all variables retained were significant at the 5% level of significance. All possible two-way interactions were created and tested for significance on by one. 

Further, the association of each dewclaw’s length with the dorsal, diagonal and heel–sole length of the corresponding weight-bearing claw was investigated in three separate mixed-effects linear regression models. Dummy variables coding for the herd of sow’s origin, sow’s parity and dewclaw’s location (medial or lateral, left or right foot, front or rear foot) were included in each of the employed initial models, which were further reduced to their final form as previously described. In addition to the fixed-effect terms, all models included a random-effect term for sow and a random-effect term for foot nested within sow to account for the hierarchical structure of multiple dewclaws length measurements on the same animal and foot.

## 3. Results

### 3.1. Claw Length Measurements

The mean values (±SD) of hoof and dewclaws dimensions in front and rear feet of sows from the three genetic lines (farms) are shown in Table 2 and Table 3 respectively. The values of all measured lengths for each of the eight hoof and dewclaw were significantly lower in samples from sows of genetic line C compared to those from sows of genetic lines B and A (*p* < 0.001). The lengths of hooves and dewclaws from sows of the genetic line B were also significantly lower compared to those from sows of the genetic line A with the exception of dorsal, diagonal and heel sole length of medial hooves in both front feet, of dorsal length of lateral hoof in front left foot and of dewclaw lengths in lateral hooves of both rear feet.

The dorsal length difference between lateral and medial hooves in front right foot was statistically lower in sows of genetic line C compared to those from sows of farms B and A, while in both rear feet it was statistically lower in sows of farm C compared only to those from sows of genetic line A.

The ratio of the dorsal length of lateral to medial hooves was statistically lower in sows of genetic line B compared to both other groups in both front feet and compared to genetic lines A and C in rear right and rear left foot respectively.

### 3.2. Macroscopic Examination

**Figure 1:**
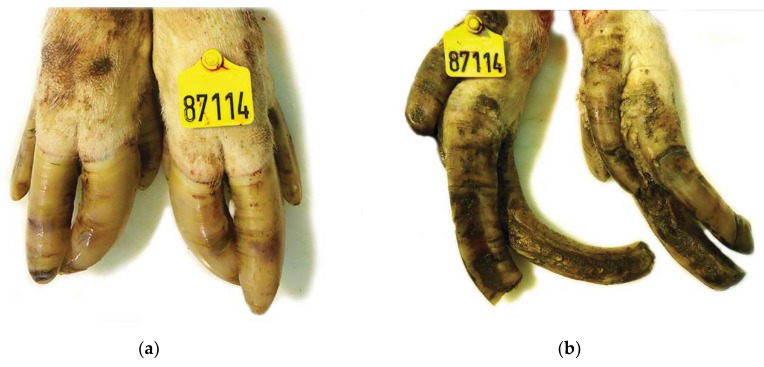
Claw lesions from a culled sow of genetic line A (PIC): (**a**) Mildly overgrown claws of the front limbs showing small cracks bruises throughout the wall hoof; (**b**) Severely overgrown and deformed rough claws of the hind limbs with hyperkeratinization, ridges, cracks and erosions near the coronary area. Overgrown dewclaws are also observed.

**Figure 2:**
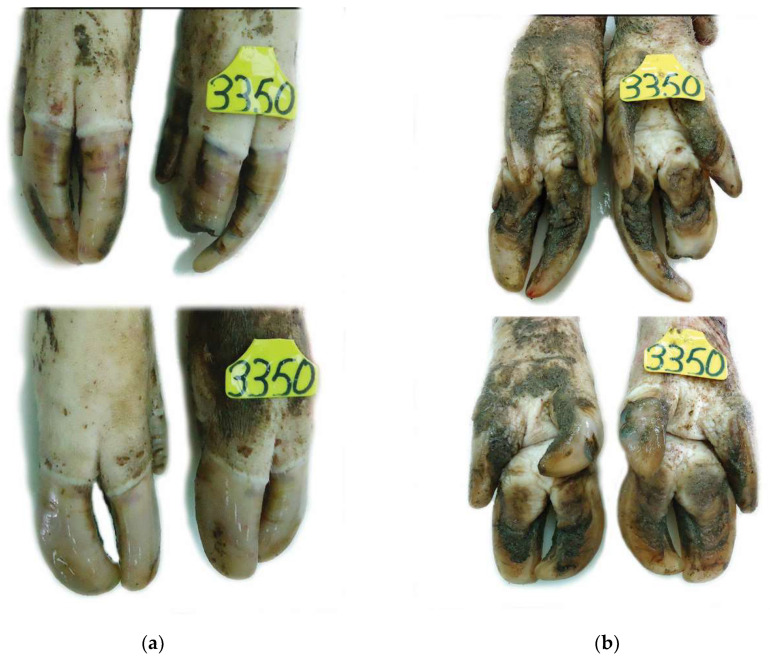
Claw lesions from a culled sow of genetic line B (Danbred): (**a**) Dorsal view: Grossly normal front limbs claws (bottom) with an intact wall and no deformity of the claw. Mildly overgrown claws of the hind limbs (top) showing superficial erosions, bruises and a ruptured hoof wall with exposure of phalange; (**b**) Ventral view: Extensive cavitation of the sole in both front and hind limbs.

**Figure 3:**
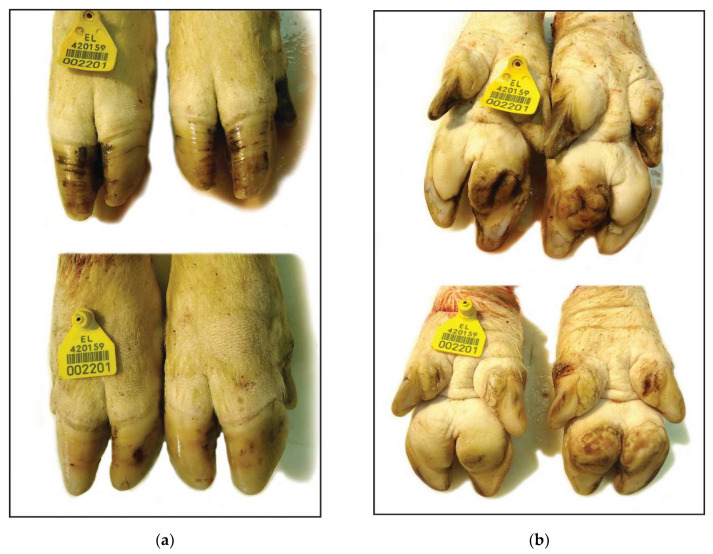
Claw lesions from a culled sow of genetic line C (Topigs): (**a**) Dorsal view: Normal length of the claws and few bruises and superficial cracks on an intact hoof wall; (**b**) Ventral view: Mild ulceration of the heel bulbs.

**Figure 4:**
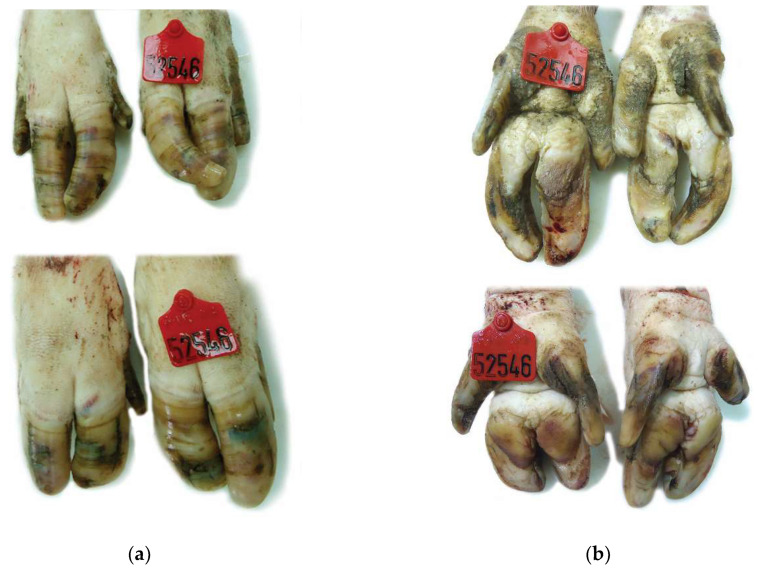
Claw lesions from a culled sow of genetic line A (PIC): (**a**) Dorsal view: Mildly overgrown claws. Multiple cracks, cavitating lesions and irregularity of the keratin are observed on the hoof wall (**b**) Ventral view: Cavitation and ulceration of the sole.

**Figure 5:**
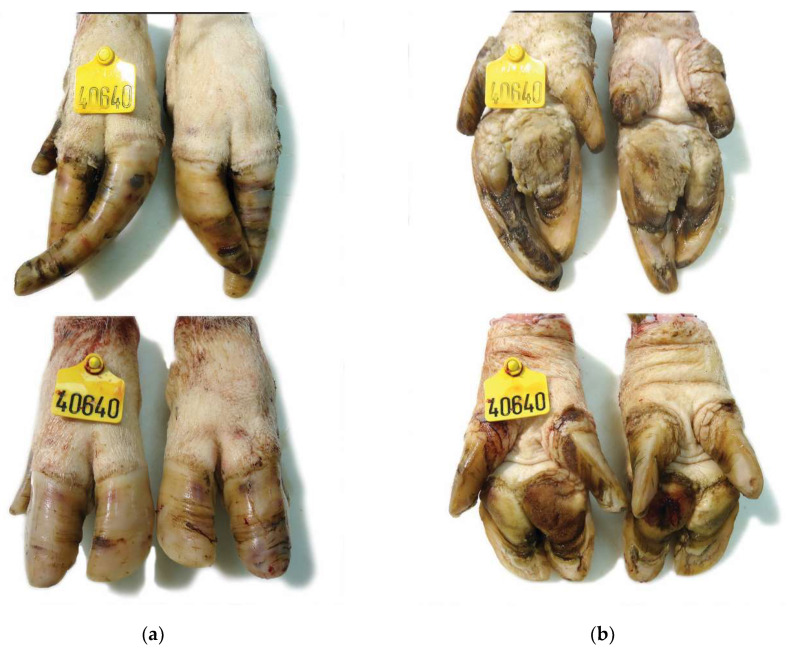
Claw lesions from a culled sow of genetic line A (PIC): (**a**) Dorsal view: Superficial cracks and erosions on the hoof wall of front limbs (bottom). Elongated and deformed claws of the hind limbs (top). The hoof wall is rough with ridges, cracks and bruises; (**b**) Ventral view: Extensive solar cavitation and hyperkeratinization and ulceration of the heel bulbs.

The frequency (%) of lesions by anatomical site and severity scale as well as the mean of the total lesion score in each hoof of sows from the three farms are presented in Table 4 and Table 5. In a total of 512 (256 front and 256 rear) hoof samples from sows of genetic line A, revealed wall, sole, white line, coronary band and heel lesions in 431 (94.6%), 398 (87,3%), 390 (85.5%), 277 (61.1%) and 228 (50.1%) samples respectively. As regards the 512 (256 front and 256 rear) hoof samples from sows of genetic line B, sole and white line lesions were detected in 335 (65.4%) and 300 (58.9%) samples respectively. Heel, wall and coronary band lesions were observed in 227 (44.3), 221 (43.2) and 142 (27.7%) samples respectively. The examination of 456 (228 front and 228 rear feet) hoof samples from sows of Farm C 339 samples (66.2%) had wall lesions while 208 (40.6%), 192 (37.5%), 167 (32.6%) and 151 (29.5) samples had white line, coronary band, heel and sole lesions respectively. In Figure 1, Figure 2, Figure 3, Figure 4 and Figure 5 description of lesions of selected claws from culled sows are provided.

### 3.3. Associations between Claw Length and Lesion Scores

In factor analyses of claw lesion scores of front and rear feet main toes, all the examined variables had Kaiser–Meyer–Olkin values > 0.5, suggesting an acceptable fit with the structure of the other variables and their suitability for inclusion in the factor analyses. The majority of the variables, from both front and rear feet claw lesion scoring, loaded highly only on a single factor, besides Factors 1 (Table 6). For Factors 1, in each of front and rear feet analysis, loaded variables representing scores of sole and white line lesions (Table 5). 

In the two final multivariable linear models (Table 7), sow parity and farm of origin retained significance while no two-way interaction was significant. SLF was associated with lesions on three foot sites on front feet, namely sole, white line and heel (Factor score 1 & Factor score 4). Particularly, for one unit increase in factor score 1 and in factor score 4, the score of the length of front feet increased by 8.3 cm (*p* < 0.001, 95% CI: 6.54; 10.05) and 2.70 cm (*p* = 0.002, 95% CI: 1.04; 4.33) on average, respectively. SLF was lower in farms C (*p* = 0.002) and B (*p* = 0.005) compared to A, but did not differ (*p* = 0.33) between C and B. SLR was associated with factor scores 1, 2, 4 and 5, representing lesions on all five examined anatomical sites of the rear feet. In detail, one unit increase in factor score 1 was accompanied by an increase of 9.64 cm (*p* < 0.001, 95% CI: 6.46; 12.82) on average in the score length of rear feet, whereas the same increase in factor 2 resulted in an average increase on score length of rear feet by 5.31 cm (*p* < 0.001, 95% CI: 2.65; 7.97). Additionally, for one unit increase on factor score 4 and 5, the score of rear feet length increased by 2.66 cm (*p* = 0.049, 95% CI: 0.01; 5.31) and 4.88 cm (*p* = 0.001, 95% CI: 2.16; 7.60) on average, respectively. SLR was lower in farm C (*p* = 0.009) compared to A and compared to B (*p* = 0.002), but did not differ (*p* = 0.78) between B and A. SDCF was associated with SLF (Pearson’s r = 0.79, *p* < 0.001) and SDCR was associated with SLR (Pearson’s r = 0.62, *p* < 0.001).

### 3.4. Associations between Length of Weight Bearing Claws and Corresponding Dewclaws 

In the three final multivariable mixed-effects linear models, left or right and medial or lateral location of dewclaws did not retained significance, while no two-way interaction was significant. In all final models, controlling for herd, parity and front or rear foot effects, dewclaw length measurements were associated with the dorsal (*p* < 0.001), the diagonal (*p* < 0.001) and the sole–heel (*p* < 0.001) length of the corresponding claw. Particularly, dewclaw length was on average longer by 0.20 cm (95% CI: 0.14; 0.25), 0.17 cm (95% CI: 0.13; 0.22) and 0.16 cm (95% CI: 0.12; 0.21) for one unit increase in the dorsal, diagonal and sole–heel length of the corresponding weight-bearing claw, respectively.

## 4. Discussion

Identifying sow culling reasons may help to resolve this issue by correcting certain practices in pig herds [30]. Sow longevity is usually decreased because of reproductive issues and lameness [31]. Sow lameness may be caused by the existence of claw lesions which may co-exist with oversized claws or dewclaws [32]. Claw lesions in sows are common in commercial herds and can reach up to 100% of the sow population [33]. In the present study, we obtained all four feet of culled sows from three commercial pig herds in order to get deeper insight into the degree of variations in lengths and lesion scores and describe their associations. Detailed claw scoring for lesions and length measurements in alive sows and at herd level is difficult to perform accurately and objectively. For this reason, a specific hydraulic chute has been used previously for inspection of sows’ claws [34]. However, such devices are not widespread in commercial pig herds and thus we decided to adjust our investigation to culled sows. Our experimental approach reveals that overgrown claws were associated with a greater lesion score estimated at different anatomical sites of the claws. Furthermore, it is shown that sows from specific genetic lines may be more prone to claw abnormalities, as the participating culled sows originated from three herds that were using three different genetic lines mostly used in Greece. To our knowledge, it is one of the few studies to report that there is a positive association between dewclaw and weight bearing claw. Thus far, dew claws have been regarded as accessory digits being under normal conditions a non-wearing claw, while the two main claws were regarded as the weight bearing digits, which normally grow and wear continuously. Our findings indicate that dew claw length was approximately 2 cm shorter than the dorsal length of the main claws. Thus, it is plausible that an area of contact between overgrown dew claws and ground may exist, which may influence the distribution of the weight bearing force applied to the sows’ feet and affect its walking pattern. We believe that the clinical importance of overgrown dew claws in conjunction with overgrown claws on overall sow claw conformation should be renegotiated. 

Evident variations among farms and by extension among genetic lines were observed in all measured claw and dewclaw lengths, in dorsal length difference between lateral and medial hooves as well as in total lesion scores. Previous findings [10] showed that chelated minerals reduced lesions but did not affect claw overgrowth. As aforementioned the sows sampled in the current study were all supplemented with the same sources of chelated minerals. Therefore, if chelates reduce lesions but not overgrowth, but as evidenced from our findings that overgrowth promotes lesions, then the positive effects of chelates on claw integrity will be surpassed by claw overgrowth which is likely genetically determined. Excessive hoof growth has been shown to have substantial genetic background in specific sow breeds. Specifically, an important degree of genetic determinism in the rate of hoof growth was detected by Quintanilla et al. [23] in females from three purebred selection lines, namely Landrace, Pietrain and Large White. The prevalence of excessive or abnormal hoof growth in Landrace and Large White females was estimated to be 36.5% and 28.2% respectively [24]. Heritabilities for hoof growth ranged from 0.24–0.27 in Landrace and from 0.36–0.38 in Large White sow populations [23,24]. Of importance was also the fact that abnormal hoof growth reduced survivability in Duroc [4,5] and Landrace sows [4]. Recently Johnson et al. [2] showed that the lateral toes in Yorkshire sows were growing slower than the lateral toes in Duroc and Crossbred (Duroc × Yorkshire) sows. 

Differences between boar breeds have been reported in regard to hoof size dissimilarity. The prevalence of uneven hooves was significantly higher in Landrace boars compared to Yorkshire and Duroc ones while the heritability estimate for this trait in hind legs was 0.61 [22]. Moreover, Fan et al. [25] identified possible genetic markers associated with hoof size asymmetry in commercial Large White and Landrace intercross females. Recently, specific hoof lesions were significantly related to boar breeds. In particular, Landrace boars showed a higher likelihood of heel overgrowth and erosion, heel sole crack and horizontal wall cracks but had a lower likelihood of overgrown hooves than Duroc boars. The probability of horizontal wall cracks, heel sole crack and dewclaw abnormality in Yorkshire boars was higher than that in Duroc boars [35]. In light of these data, findings of the present study may indicate a genetic basis behind claw abnormalities and highlight the importance of genetic selection for improved claw quality.

It should be noted that hoof trimming was not practiced in the participating herds in the present study and it is rarely practiced in pig herds in Greece. Claw trimming improved gait and locomotion in sows [32]. Changing the formation of the angle of the claw led to a better stability of sows and reduced chances of slipping [32]. Thus, it is plausible that sows with overgrown hooves and co-existence of lesions as in the current study, differences on gait and/or weight bearing capacity on each leg and increased risk of slipping may have occurred as well. Additionally, differences between farms occurred for the dorsal length difference between lateral and medial hooves which were more evident in the rear feet of sows from genetic line A compared to B and C. Most probably, the observed length difference between lateral and medial hooves may compromise locomotor ability of sows and predispose to lameness. According to van Amstel et al. [15], claw size difference on the rear feet together with claw horn lesions, showed that a possible laterality in weight bearing capacity between the exterior and the interior claw of the rear feet. The underlying reasons for the observed differences on the length of hooves between genetic lines from the three different farms is not evident and could be attributed to several factors. It is known that claw horn production is a result of a complex physiological procedure involving multiplication, keratinization and death of keratinocytes [36,37]. The synthesis of horn is affected by numerous dietary factors such as amino acids, fatty acids, minerals and vitamins [37]. In case of insufficient nutrient supply, the horn production will be disturbed [37]. It has been suggested that inadequate nutrient supply may be caused due to local increase of the mechanical pressure [37,38]. Several other mechanisms were proposed such as shifts in metabolism at farrowing and lactation period and the involvement of active biomolecules such as histamine [37,38,39]. Type of flooring affects also hoof quality and growth, as in solid flooring hoof lesions were observed more often compared to slatted floors [40]. Further research is warranted to elucidate the underlying mechanisms for the differences in claw lengths between different genetic lines.

On farm assessment of sows’ hoof lesions has been the subject of previous research [8,9,10]. In the current study the most frequent lesions detected in the total of 1480 hooves were wall, white line and sole lesions. High incidence of wall cracks was also reported by Anil et al. [41], Pluym et al. [7] and Varagka et al. [13] and was associated with high sow parity [7]. White line was also found elsewhere [13,41] to be an area frequently affected. Concurrent lesions on the white line and sole areas in the present study could be ascribed to the fact that these two anatomical are adjacent [8]. It is known that multiparous sows, have increased metabolic requirements to cover their reproductive performance [42]. Especially towards the end of gestation and during lactation, metabolic demands are increased and, therefore, any reduced nutrient supply could lead to a compromised claw quality [42]. Therefore, other factors than the genetic line may have also contributed to the increased hoof lesion scored observed in sows from genetic line A in the present study. 

The detection of statistically significant correlation between almost all claw lengths and lesion score, reinforces the opinion that overgrowth confers changes in the weight-bearing pattern between and within hooves, rendering some anatomical areas, especially caudal ones, more prone to injuries due to overloading [43]. A farm-genetic line effect was detected for the correlations between the sum of length of measurements in both front and rear feet with claw lesion scores being lower in genetic line C and B than A and in C than A and B, respectively. It has been suggested that under field conditions, claw lesions incidence is variable between herds and also between countries [19,42]. Calderón Díaz et al. [17] demonstrated that sows with overgrown hooves had higher scores for toe, heel and sole erosion. Our findings corroborate with those of Sasaki et al. [19], who showed an association between claw lesion severity and claw lengths. It was proposed that the sole length in the lateral claws could be used to estimate claw lesions. In the latter study however, only hind limb claw lesions and lengths were measured. In our study, all four feet of culled sows were investigated. The sum of length measurements in the front feet were associated with lesions in sole, white line and heel of front feet, while the sum of length of measurements in the rear feet were associated with lesions in all five anatomical sites investigated (sole, white line, heel, wall, coronary band). This reveals that severity of lesions may be more increased in the rear feet than the front ones, which could be attributed to a difference in weight-bearing distribution towards the hind legs in a mature sow. Nevertheless, the present findings show that front feet claws may also suffer from lesions. Compared to Sasaki et al. [19], in our study lesions were present not only in the weight bearing elements of rear feet (heel, sole) but also on anatomical sites in the dorsal surface of the toes (wall, coronary band). Considering the positive association between claw lesions and overall claw length measurements, it could be suggested that a robust claw is one with compact dimensions. In overgrown claws the quality of the horn tissue produced may be compromised, probably affecting its mechanical properties and elasticity. This field warrants further investigation.

## 5. Conclusions

The present study showed that claw overgrowth and claw lesions are a common problem in Greece with evident variations between genetic lines. Dorsal length difference between lateral and medial hooves especially in the rear feet differed significantly between farms, while the lengths of the main toes were correlated with the length of dew claws on front and rear feet. The sum of length measurements of the main toes of the front feet (SLF) associated with lesions on sole, white line and heel of front feet, while sum of length measurements of the main toes of the rear feet (SLR) associated with all lesions of the rear feet. Overall, sows’ claw lesion severity and claw lengths may differ between genetic lines and frequency of lesions is higher in longer claws. Further research is needed towards elucidating whether in overgrown claws the quality of the horn tissue is affected by altering its mechanical properties and elasticity.

## Figures and Tables

**Table 1:** Scoring system applied for evaluation of lesions.

Anatomical Site	Score 0	Score 1	Score 2
Sole	No lesions or very small superficial cracks in the epidermis	Serious lesions in the epidermis not extending into the corium, heel–sole separation, or both	One or more deep cracks extending into the corium, severe heel–sole separation, or both
Heel	No lesions or very small superficial cracks in the epidermis	Hyperkeratinization and erosions in the epidermis not extending into the corium	Hyperkeratinization, deep cracks extending into the corium and often necrosis
White line	No lesions or very small superficial cracks in the epidermis	Wall-sole separation not extending into the corium	Wall-sole separation extending into the corium
Wall	No lesions or very small superficial cracks in the epidermis	Cracks not extending into the corium, often accompanied by bruising	Cracks extending into the corium, separation of the keratin, or both
Coronary band	No lesions or very small superficial cracks in the epidermis	Edema with purulent exudate, hemorrhage and necrosis, or both	NA

NA = not applicable; for the coronary band, lesion score was 0 when healthy and 1 when any lesion was observed.

**Table 2:** Claw lengths: Dorsal, the distance from the dorsal skin-horn junction (periople) to the apex of the toe; Diagonal, the distance from the apex of the toe to the skin-horn junction at the heel; Heel-Sole, the length of the abaxial wall (sole) and bulb (heel) that are in contact with the floor surface from the top of the toe to the caudal end of the heel; Dewclaw (along dorsal wall from just below the coronary band to the end of the wall); Dorsal length difference between lateral and medial hooves, expressed as mean ± SD of culled sows’ front feet from three pig farms (A, B, C). The sows of each farm belonged to different genetic lines (Farm A: PIC; Farm B: Danbred; Farm C: Topigs).

		Genetic Lines-Farms		
	A	B	C	*p*-Value
**Front right foot**				
Medial claw lengths, cm				
Dorsal	6.16 ± 1.34 ^a^	5.87 ± 1.10 ^a^	4.61 ± 0.47 ^b^	<0.001
Diagonal	8.00 ± 1.48 ^a^	7.56 ± 1.06 ^a^	6.43 ± 0.57 ^b^	<0.001
Heel-Sole	9.07 ± 1.44 ^a^	8.55 ± 1.14 ^a^	7.48 ± 0.60 ^b^	<0.001
Dewclaw	4.63 ± 1.29 ^a^	3.85 ± 0.86 ^b^	3.19 ± 0.39 ^c^	<0.001
Lateral claw lengths, cm				
Dorsal	6.19 ± 1.40 ^a^	5.71 ± 1.15 ^b^	4.73 ± 0.55 ^c^	<0.001
Diagonal	7.98 ± 1.51 ^a^	7.34 ± 1.19 ^b^	6.39 ± 0.53 ^c^	<0.001
Heel-Sole	9.32 ± 1.61 ^a^	8.55 ± 1.26 ^b^	7.66 ± 0.60 ^c^	<0.001
Dewclaw	4.71 ± 1.01 ^a^	4.29 ±1.16 ^b^	3.53 ± 0.48 ^c^	<0.001
Dorsal length difference between lateral and medial hooves, cm	0.51 ± 0.53 ^a^	0.46 ± 0.54 ^a^	0.26 ± 0.19 ^b^	0.009
**Front left foot**				
Medial claw lengths, cm				
Dorsal	6.04 ± 1.23 ^a^	5.94 ± 1.64 ^a^	4.51 ± 0.48 ^b^	<0.001
Diagonal	7.91 ± 1.33 ^a^	7.69 ± 1.53 ^a^	6.51 ± 0.53 ^b^	<0.001
Heel-Sole	8.68 ± 1.28 ^a^	8.55 ± 1.66 ^a^	7.27 ± 0.59 ^b^	<0.001
Dewclaw	4.38 ± 1.10 ^a^	3.84 ± 1.10 ^b^	3.14 ±0.45 ^c^	<0.001
Lateral claw lengths, cm				
Dorsal	6.24 ± 1.25 ^a^	5.91 ± 1.35 ^a^	4.78 ±0.56 ^b^	<0.001
Diagonal	7.81 ± 1.25 ^a^	7.39 ± 1.46 ^b^	6.30 ± 0.48 ^c^	<0.001
Heel-Sole	9.40 ± 1.42 ^a^	8.76 ± 1.46 ^b^	7.75 ± 0.67 ^c^	<0.001
Dewclaw	5.10 ± 1.11 ^a^	4.19 ± 0.97 ^b^	3.58 ± 0.56 ^c^	<0.001
Dorsal length difference between lateral and medial hooves, cm	0.51 ± 0.52	0.52 ± 0.83	0.31 ± 0.22	0.133

^a,b,c^ Mean values within the same row with different superscript letter are significantly different (*p* < 0.05).

**Table 3:** Claw lengths: Dorsal, the distance from the dorsal skin-horn junction (periople) to the apex of the toe; Diagonal, the distance from the apex of the toe to the skin-horn junction at the heel; Heel–Sole, the length of the abaxial wall (sole) and bulb (heel) that are in contact with the floor surface from the top of the toe to the caudal end of the heel; Dewclaw (along dorsal wall from just below the coronary band to the end of the wall); Dorsal length difference between lateral and medial hooves, expressed as mean ± SD of culled sows’ rear feet from three pig farms (A, B, C). The sows of each farm belonged to different genetic lines (Farm A: PIC; Farm B: Danbred; Farm C: Topigs).

		Genetic Lines-Farms		
	A	B	C	*p*-Value
Rear right foot				
Medial claw lengths, cm				
Dorsal	7.22 ± 2.21 ^a^	6.04 ± 1.32 ^b^	4.84 ± 0.56 ^c^	<0.001
Diagonal	8.95 ± 2.49 ^a^	7.33 ± 1.23 ^b^	6.22 ± 0.53 ^c^	<0.001
Heel-Sole	10.35 ± 2.83 ^a^	8.47 ± 1.31 ^b^	7.24 ± 0.65 ^c^	<0.001
Dewclaw	5.95 ± 1.62 ^a^	4.69 ± 1.05 ^b^	3.89 ± 0.68 ^c^	<0.001
Lateral claw lengths, cm				
Dorsal	8.17 ± 2.59 ^a^	6.40 ± 1.49 ^b^	5.35 ± 0.71 ^c^	<0.001
Diagonal	10.03 ± 2.87 ^a^	7.88 ± 1.52 ^b^	6.76 ± 0.69 ^c^	<0.001
Heel-Sole	11.46 ± 2.93 ^a^	9.15 ± 1.53 ^b^	7.95 ± 0.90 ^c^	<0.001
Dewclaw	5.38 ± 1.37 ^a^	4.86 ± 1.08 ^a^	3.76 ± 0.55 ^b^	<0.001
Dorsal length difference between lateral and medial hooves, cm	2.02 ± 2.21 ^a^	0.75 ± 0.76 ^b^	0.53 ± 0.44 ^b^	<0.001
Rear left foot				
Medial claw lengths, cm				
Dorsal	7.33 ± 1.84 ^a^	5.89 ± 1.10 ^b^	4.83 ± 0.57 ^c^	<0.001
Diagonal	8.98 ± 2.06 ^a^	7.28 ± 1.11 ^b^	6.38 ± 0.53 ^c^	<0.001
Heel-Sole	10.09 ± 2.08 ^a^	8.19 ± 1.23 ^b^	7.06 ± 0.64 ^c^	<0.001
Dewclaw	5.28 ± 1.46 ^a^	4.56 ± 0.98 ^b^	4.00 ± 0.80 ^c^	<0.001
Lateral claw lengths, cm				
Dorsal	7.66 ± 2.00 ^a^	6.18 ± 1.17 ^b^	5.36 ± 0.92 ^c^	<0.001
Diagonal	9.23 ± 2.40 ^a^	7.62 ± 1.17 ^b^	6.66 ± 0.99 ^c^	<0.001
Heel-Sole	10.84 ± 2.43 ^a^	8.91 ± 1.16 ^b^	8.09 ± 1.20 ^c^	<0.001
Dewclaw	5.48 ± 1.78 ^a^	4.93 ± 1.17 ^a^	3.96 ± 0.83 ^b^	<0.001
Dorsal length difference between lateral and medial hooves, cm	1.48 ± 1.75 ^a^	0.64 ± 0.70 ^b^	0.58 ± 0.56 ^b^	<0.001

^a,b,c^ Mean values within the same row with different superscript letter are significantly different (*p* < 0.05).

**Table 4:** Percentage of medial and lateral claws of front feet in each of the 3 classes of lesion scores on five foot sites (sole, heel, white line, wall, coronary band) in slaughtered sows from three pig farms (A, B, C). The sows of each farm belonged to different genetic lines (Farm A: PIC; Farm B: Danbred; Farm C: Topigs).

			Sole			Heel			White Line			Wall			Coronary Band	
Lesion Score			0	1	2	0	1	2	0	1	2	0	1	2	0	1
Right	Medial	A	24.6	47.4	28.1	70.2	24.6	5.3	17.5	68.4	14.0	5.3	70.2	24.6	45.6	54.4
		B	31.3	51.6	17.2	54.7	40.6	4.7	48.4	43.8	7.8	65.6	34.4	0.0	84.4	15.6
		C	85.9	14.1	0.0	79.7	20.3	0.0	76.6	23.4	0.0	40.6	59.4	0.0	67.2	32.8
	Lateral	A	14.0	57.9	28.1	57.9	36.8	5.3	19.3	66.7	14.0	3.5	63.2	33.3	43.9	56.1
		B	37.5	46.9	15.6	62.5	34.4	3.1	50,0	42.2	7.8	64.1	32.8	3.1	87.5	12.5
		C	78.1	20.3	1.6	64.1	35.9	0.0	68.8	31.3	0.0	39.1	57.8	3.1	75.0	25.0
Left	Medial	A	12.3	63.2	24.6	59.6	38.6	1.8	29.8	59.6	10.5	3.5	73.7	22.8	52.6	47.4
		B	35.9	43.8	20.3	54.7	40.6	4.7	39.1	48.4	12.5	64.1	34.4	1.6	71.9	26.6
		C	81.3	18.8	0.0	81.3	18.8	0.0	73.4	25.0	1.6	37.5	62.5	0.0	53.1	46.9
	Lateral	A	19.3	54.4	26.3	47.4	43.9	8.8	17.5	71.9	10.5	5.3	66.7	28.1	45.6	54.4
		B	34.4	48.4	17.2	60.9	35.9	3.1	34.4	53.1	12.5	68.8	31.3	0.0	84.4	15.6
		C	79.7	18.8	1.6	65.6	34.4	0.0	71.9	23.4	4.7	40.6	59.4	0.0	60.9	39.1

0: no lesions; 1: erosions (sole, heel). Seperation (white line), bruises (wall). Any kind of lesions (coronary band); 2: ulcers (sole, heel), deep seperation (white line), cracks (wall).

**Table 5:** Percentage of medial and lateral claws of rear feet in each of the 3 classes of lesion scores on five foot sites (sole, heel, white line, wall, coronary band) in slaughtered sows from three pig farms (A, B, C). The sows of each farm belonged to different genetic lines (Farm A: PIC; Farm B: Danbred; Farm C: Topigs).

			Sole			Heel			White Line			Wall			Coronary Band	
Lesion Score			0	1	2	0	1	2	0	1	2	0	1	2	0	1
Right	Medial	A	71.9	23.4	4.7	82.8	15.6	1.6	56.3	40.6	3.1	35.9	57.8	6.3	62.5	37.5
		B	45.3	45.3	9.4	82.8	15.6	1.6	45.3	51.6	3.1	60.9	34.4	4.7	75.0	25.0
		C	10.7	44.6	44.6	58.9	32.1	8.9	14.3	51.8	33.9	8.9	30.4	60.7	26.8	73.2
	Lateral	A	39.1	51.6	9.4	42.2	48.4	9.4	31.3	64.1	4.7	28.1	54.7	17.2	54.7	45.3
		B	25.0	56.3	18.8	32.8	59.4	7.8	28.1	60.9	10.9	40.6	56.3	3.1	62.5	37.5
		C	3.5	19.3	77.2	17.5	50.9	31.6	1.8	42.1	56.1	0.0	31.6	68.4	15.8	84.2
Left	Medial	A	79.7	17.2	3.1	84.4	12.5	3.1	56.3	40.6	3.1	23.4	73.4	3.1	65.6	34.4
		B	46.9	43.8	9.4	68.8	28.1	3.1	56.3	40.6	3.1	46.9	48.4	4.7	53.1	46.9
		C	14.0	54.4	31.6	71.9	19.3	8.8	14.0	63.2	22.8	12.3	56.1	31.6	45.6	54.4
	Lateral	A	48.4	39.1	12.5	39.1	53.1	7.8	40.6	50.0	9.4	25.0	65.6	9.4	60.9	39.1
		B	20.3	60.9	18.8	28.1	65.6	6.3	29.7	62.5	7.8	43.8	46.9	9.4	59.4	40.6
		C	3.6	29.1	67.3	16.4	58.2	25.5	1.8	52.7	45.5	1.8	51.8	46.4	35.7	64.3

0: no lesions; 1: erosions (sole, heel). Seperation (white line), bruises (wall). Any kind of lesions (coronary band); 2: ulcers (sole, heel), deep seperation (white line), cracks (wall).

**Table 6:** Factor scores, with loadings > |0.4,|, produced from factor analyses of claw lesion scores of front and rear feet main toes of 185 culled sows from three Greek herds, examined for association with the score for the sum of length measurements of the main toes of the front feet and the score for the sum of length measurements of the main toes of the rear feet.

	Factor Score	Claw Lesion Scores
Factor analysis of front feet claw lesions	Factor score 1	For sole and white line lesions on the front right and on the front left foot
	Factor score 2	For wall lesions on front right and the front left foot
	Factor score 3	For coronary band lesions on front right and the front left foot
	Factor score 4	For heel lesions on front right and the front left foot
Factor analysis of rear feet claw lesions	Factor score 1	For sole and white line lesions on the rear right and on the rear left foot
	Factor score 2	For wall lesions on rear right and the rear left foot and coronary band lesions on the rear right foot
	Factor score 3	For heel lesions on rear left medial and on rear right medial claws
	Factor score 4	For coronary band lesions on rear left medial and on rear left lateral claws
	Factor score 5	For heel and white line lesions on the rear left lateral claw

**Table 7:** The two final multivariable linear regression models for the association between the score for the sum of length measurements of the main toes of the front feet (SLF) and the score for the sum of length measurements of the main toes of the rear feet (SLR) of 185 culled sows from three Greek herds, with the produced standardized factor scores after factor analyses of claw lesion scores of front and rear feet main toes, adjusted for parity and herd- genetic line effect.

	SLF	SLR				
	Coef.	*p*-Value	95% C.I.	Coef.	*p*-Value	95% C.I.
constant	87.00	<0.001	82.17; 91.83	98.60	<0.001	89.53; 107.68
herd	−3.73	0.001	−5.84; −1.62	−6.43	0.002	−10.37; −2.50
parity	1.18	<0.001	0.62; 1.75	1.40	0.001	0.56; 2.24
Factor score 1 ^f^	8.30	<0.001	6.54; 10.05	NA		
Factor score 2 ^f^	-	-	-	NA		
Factor score 3 ^f^	-	-	-	NA		
Factor score 4 ^f^	2.70	0.002	82.17; 91.83	NA		
Factor score 1 ^r^	NA			9.64	<0.001	6.46; 12.82
Factor score 2 ^r^	NA			5.31	<0.001	2.65; 7.97
Factor score 3 ^r^	NA			-	-	-
Factor score 4 ^r^	NA			2.66	0.049	0.01; 5.31
Factor score 5 ^r^	NA			4.88	0.001	2.15; 7.60

^f^ produced factor score after factor analysis for claw lesion scores in front feet; ^r^ produced factor score after factor analysis for claw lesion scores in rear feet; NA: not applicable.

## Data Availability

Data are available from corresponding author upon request.
